# Becoming an Open Dialogue practitioner: a qualitative study of practitioners’ training experiences and transitioning to practice

**DOI:** 10.3389/fpsyg.2024.1432327

**Published:** 2024-08-12

**Authors:** Eleftherios Anestis, Timothy Weaver, Claire Melia, Katherine Clarke, Steve Pilling

**Affiliations:** ^1^Department of Mental Health and Social Work, Faculty of Health, Social Care & Education, Middlesex University London, London, United Kingdom; ^2^Government Social Research, UK Civil Service, London, United Kingdom; ^3^Department of Clinical, Educational and Health Psychology, Division of Psychology and Language Sciences, University College London, London, United Kingdom

**Keywords:** Open Dialogue, mental health, open dialogue training, staff perspectives, transformational learning

## Abstract

**Introduction:**

In the context of transforming mental healthcare towards more personalised and recovery-oriented models, Open Dialogue has attracted significant international interest. Open Dialogue proposes a way of organising services and delivering care that supports an immediate response to crisis, relational continuity of care, a social network approach and the empowerment of networks through shared decision-making and a flattened hierarchy. The ODDESSI trial currently being conducted in the UK is assessing the model’s clinical and cost-effectiveness. Practitioners who delivered the approach within the trial undertook a one-year Open Dialogue foundation training programme, however little is known about their training experiences. This study aimed to explore practitioners’ experiences of receiving the training and transitioning to dialogic practice.

**Methods:**

Individual, joint and focus group interviews with 32 Open Dialogue practitioners were conducted. Thematic analysis was used to analyse the transcripts and transformational learning theory informed the interpretation of the findings.

**Results:**

Two themes further divided in subthemes were generated from the data: (1) experiences and impact of formal training and (2) becoming an Open Dialogue practitioner as an ongoing learning process beyond formal training: barriers and facilitators.

**Discussion:**

The one-year Open Dialogue foundation training was a transformative experience for participants due to its emphasis on self-work and its impact on a personal level. Practitioners felt adequately prepared by their training for dialogic practice, yet becoming an OD practitioner was seen as a continual process extending beyond formal training, necessitating ongoing engagement with the approach and organisational support. However, the commitment of participants to deliver optimal dialogic care was occasionally impeded by organisational constraints, resource limitations, and often having to concurrently deliver conventional care alongside Open Dialogue.

## Introduction

Responding to challenges in providing high quality and timely mental healthcare whilst also tackling inequalities in access and outcomes, England’s National Health Service (NHS) Mental Health Implementation Plan stressed the need for a transformation of mental health services (NHS England, 2019). Amongst its objectives, the plan supports the development of new integrated models of crisis and community care for people with severe mental illnesses, with personalised approaches that give service users more control and choice over their care. This is aligned with the World Health Organisation Comprehensive Mental Health Action Plan 2013–2030 that urges the importance of the development of responsive, comprehensive and integrated community-based mental health services that promote human rights and recovery. In England, promoting personal recovery, that is supporting people to achieve ‘a way of living a satisfying, hopeful and contributing life even with the limitations caused by illness’ (Anthony, 1993, p.17) has been one of the priorities for public mental health policy (Coffey et al., 2019). However, in the care of people with severe mental illnesses, the translation of the principle of personal recovery into practice has proved to be challenging and sporadic in the context of organisations and professionals which prioritise the control of symptoms and risk mitigation over supporting social integration (Stasiulis et al., 2022). This suggests that for a transformation of mental health services that is genuinely recovery-focused, new ways of providing care to people with severe mental illnesses should be considered.

Open Dialogue (OD) can be considered as an appropriate approach to respond to the objectives mentioned above, being a recovery-oriented and human rights-aligned (von Peter et al., 2019) model of mental health care. OD aims to empower networks to achieve and maintain recovery from a mental health crisis through a collaborative effort between a multidisciplinary team, the service-user and their network (Seikkula and Olson, 2003). It supports an immediate response to a person experiencing a mental health crisis (within 24 h) through ‘network meetings’ which are organised by a multidisciplinary healthcare team trained in OD. The time, place, duration and frequency of these meetings are established by the network and the professionals involved in the first meeting should remain the same throughout the duration of the treatment to ensure relational continuity of care. Unlike other approaches that favour professionals’ expertise and a directive approach, OD emphasises the importance of generating dialogue and collaboration between the service-user, their informal family and social network and the professional team characterised by a flattened hierarchy (Seikkula et al., 2006). During network meetings, OD practitioners avoid rushing into a diagnosis or conclusions and are expected to have greater tolerance of uncertainty in terms of risk. The main role of practitioners is to foster an inclusive dialogue wherein every member of the network contributes (polyphony), with the aim to cultivate an intersubjective understanding of the crisis and promote shared decision-making (Olson et al., 2014). In this context, psychiatric knowledge and treatments may be utilised but in a more person-centred manner that respects service users’ perspectives, voiced needs and preferences. The approach can be summarized through seven principles: immediate help, social network perspective, flexibility and mobility, responsibility, psychological continuity, tolerance of uncertainty and dialogism (Seikkula et al., 2006).

A review of the available evidence on the effectiveness of OD as an intervention revealed promising findings such as symptom reduction and reduced hospitalisation rates (Freeman et al., 2019). However, out of the 23 studies included in the review, 16 were qualitative, whilst the remaining quantitative studies had small sample sizes, lacked randomisation and control group comparison. Only three of the studies were conducted outside Scandinavia. The authors, therefore, argued that no strong conclusions can be made regarding the efficacy of OD and that future research should focus on how OD can be scaled and implemented outside Scandinavia (Buus et al., 2021). They emphasised the need for a Randomised Controlled Trial (RCT) to assess the effectiveness of OD compared to routine care. The ODDESSI (Open Dialogue: Development and Evaluation of a Social Network Intervention for Severe Mental Illness) research programme does precisely this. Conducted in the UK, it includes an RCT examining the clinical and cost effectiveness of OD as a crisis intervention in comparison to treatment as usual (TAU) (Pilling et al., 2022). The trial also includes an embedded qualitative process evaluation. The findings presented in this paper represent a partial analysis of process evaluation data obtained from the practitioners who delivered OD as part of the trial.

In preparation for the ODDESSI trial, multidisciplinary teams (including peer practitioners) from six participating NHS sites took part in a one-year foundation training in OD. In general, prolonged exposure to training, varied training techniques, ongoing follow-up support, supervision, and increased motivation can affect the transfer of knowledge from mental health training to practice (Lyon et al., 2011; McGonagle and Jackson, 2017). An organisational environment that provides opportunities to practise these skills can also play a crucial role (McGonagle and Jackson, 2017). Unlike other approaches, OD does not offer a manualized training curriculum (Buus et al., 2022a). Because of its focus on professionals ‘being with’ others in crisis and facilitating dialogue, OD training is usually characterised by an experiential, interactive and reflective approach to learning. The original training in Western Lapland in Norway is a three-year family therapy programme informed by the principles of systemic family therapy, the need-adapted approach and the reflective approach (Aaltonen et al., 2011). OD training programmes around the world differ in terms of their duration and nature, but personal development through self-work is considered the main aim of the training (Stockmann et al., 2019a; Jacobsen et al., 2021; Buus et al., 2022b). In Norway, Jacobsen et al. (2021) investigated professionals’ experiences of a shorter 6-day training delivered over six months and found that it was successful in achieving its learning outcomes and increasing trainees’ confidence in delivering OD. In a focus group study in the UK, 13 healthcare professionals discussed their experiences of completing a three-year OD training programme. Examining the topic through a transformational learning lens (Mezirow, 2003), the authors suggested that OD training fostered a process of critical reflection and self-exploration, with participants reporting personal changes and deeper relationships with others as outcomes of the training (Wates et al., 2022). Similarly, a longitudinal focus-group study in Australia examined the learning processes and therapeutic practice of healthcare professionals who completed a two or three-year advanced OD training programme (Buus et al., 2022a). In this study, participants talked about the transformative impact of the training on a personal level but also discussed how they have incorporated the principles of OD in their practice in a flexible and less dogmatic way.

Building on the findings from these studies, the aim of the current study was to explore the OD training experiences of healthcare professionals who delivered OD within the ODDESSI trial. Most of these practitioners attended a year-long OD foundation training programme spread across four residential weeks, whilst some attended the same training remotely due to the COVID-19 pandemic. More information about the nature of this training as it was experienced by participants can be found in the results section of this paper. These professionals were expected to deliver OD to high standards of care demonstrating fidelity of OD service delivery (Olson et al., 2014) and adherence to the principles of dialogic practice (Lotmore et al., 2023). Exploring their training experiences is therefore crucial in the future contextualisation and interpretation of the trial outcomes. The aim of the current study is to investigate OD practitioners’ experiences of the one-year OD foundation training and transitioning to practice within the ODDESSI trial.

## Methods

### Study design

This paper is based on a partial analysis of data from a qualitative process evaluation which was implemented as part of the ODDESSI trial. The process evaluation included qualitative sub-studies examining practitioners’ and service users’ experiences of delivering and receiving OD and TAU, respectively, (to be reported elsewhere). The data and findings presented in this paper are based on an analysis of a subset of in-depth interviews and focus groups with OD practitioners which explored their perspectives on the training received, their preparedness to deliver OD, and finally their post-training experiences in applying OD principles and developing their OD practice through their work with service users.

### Participants and recruitment

Participants were all OD practitioners recruited from the six different NHS trusts which took part in the ODDESSI trial. Practitioners were part of OD teams that were formed for the purpose of the trial. At one NHS trust practitioners practised OD only, but at other sites they also delivered TAU to service users not enrolled on the trial apart. Practitioners who had received OD training and practised OD at the time of the interview were eligible for inclusion. Using lists of OD staff working at each site we employed a purposive sampling strategy to achieve range and diversity in terms of participating site and staff role. There were no exclusion criteria in terms of length of experience, age or professional background. Potential participants received a participant information sheet via email prior to the interview and informed consent was obtained before the start of each interview. In total, 32 OD practitioners were recruited in this study. Participants’ characteristics can be found in Table 1, however, considering the limited number of OD practitioners that were part of the trial, place of work, sex and age were not included on the table to reduce the risk of identification. Overall, we recruited 20 female and 12 male participants, 12 nurses, 7 peer practitioners, 5 consultant psychiatrists, 3 occupational therapists, 2 social workers, 1 clinical psychologist and 2 support workers. Participants from all six NHS sites were recruited, with most practitioners (*n* = 19) having a TAU caseload apart from their OD caseload.

**Table 1:** Participants’ characteristics.

Participant number	Role	Type of training	TAU caseload
P1	Social Worker	Residential	Yes
P2	Nurse	Residential	No
P3	Nurse	Residential	Yes
P4	OD Practitioner	Residential	No
P5	Nurse	Residential	Yes
P6	Peer practitioner	Residential	No
P7	Nurse	Residential	Yes
P8	Nurse	Residential	No
P9	Nurse	Residential	Yes
P10	Peer practitioner	Residential	No
P11	Occupational Therapist	Residential	No
P12	Nurse	Online	Yes
P13	Occupational Therapist	Residential	No
P14	Consultant Psychiatrist	Residential	Yes
P15	Consultant Psychiatrist	Residential	Yes
P16	Occupational Therapist	Residential	No
P17	Consultant Psychiatrist	Residential	Yes
P18	Consultant Psychiatrist	Residential	Yes
P19	Nurse	Residential	Yes
P20	Peer Practitioner	Residential	No
P21	Nurse	Residential	No
P22	Nurse	Residential	Yes
P23	Nurse	Online	Yes
P24	Peer Practitioner	Online	No
P25	Nurse	Residential	Yes
P26	Peer Practitioner	Residential	Yes
P27	Peer Practitioner	Residential	Yes
P28	Peer Practitioner	Residential	No
P29	Social Worker	Residential	No
P30	Clinical Psychologist	Residential	Yes
P31	Consultant Psychiatrist	Residential	Yes
P32	Support Worker	Online	Yes

### Data collection and analysis

Data were collected through individual interviews (*n* = 16) and joint interviews with 2 respondents (*n* = 3) and a small number of focus groups (*n* = 3). The data collection phase started in June 2020 and finished in April 2021. For most of this period, the UK was under a national COVID-19 lockdown. Hence, data collection could only be completed online. This was a challenging time for services. Due to staff re-deployment, sickness or workload issues, some opportunistic sampling was required based on staff availability. Similarly, we took a pragmatic approach to data collection method and where circumstances required we interviewed staff jointly or in small focus groups.

All interviews and focus groups were conducted using topic guides which were informed by a literature review and an *a priori* logic model. This logic model was developed to describe the model of OD evaluated by the trial, showing how its intended organisational and process characteristics, and the actions (and interaction) of critical practice-based ingredients of care, were intended to affect the hypothesised trial outcomes. In their totality, the topic guides used with practitioners (and indeed trial participants) within the OD study arm were designed to interrogate the working of the logic model. Once initially drafted, the topic guides were further amended through consultation with the trial team and the project’s Lived Experience Advisory Panel. Topic guides were also refined progressively during the study, reflecting discussions and emerging themes from interviews. A list of the questions related to the scope of this study can be found as a Supplementary material.

Interviews were audio recorded and transcribed verbatim. We analysed the data using thematic analysis following the methods outlined by Braun and Clarke (2006, 2019). EA familiarised himself by reading the transcripts once before moving on to the analysis process. Data that related to the research question were coded following an inductive approach as an attempt to closely represent participants’ experiences of receiving OD training and becoming an OD practitioner. However, a both inductive and deductive approach was followed in the generation of themes, as transformational learning theory influenced the interpretation and conceptualization of participants’ experiences. Transformational learning is an adult learning theory that describes a four-stage process of deep learning beyond knowledge and skills acquisition. Learners who engage in transformational learning (a) encounter knowledge that challenges their beliefs and values, (b) engage in critical reflection of their assumptions and (c) provisionally try new roles which eventually leads to (d) transformative change in their worldviews and behaviour (Mezirow, 1991). Themes and subthemes were generated by going back and forth to the codes and via discussions between EA and TW, with the resulting themes considered effective in reflecting participants’ experiences in a coherent and insightful manner. Reflective processes were an integral part of the analysis, making sure that discussions took place before making any decision throughout the analytic process. Our shared goal was to generate both an authentic representation of participants’ experiences by setting aside preconceptions on the topic but also findings with meaningful implications for further research and implementation.

## Results

Two themes further divided in subthemes were generated from the data: (1) experiences and impact of formal training and (2) becoming an OD practitioner as an ongoing learning process beyond formal training: barriers and facilitators. Before presenting these two themes, a summary of the nature of the training as it was described by participants will be provided which will help contextualise the findings of the study.

### Nature of training

Participants in this study represent different training cohorts that received different types of the training: most (*n* = 28) received a year-long training spread across four residential weeks whilst four participants received an online version of the training due to the COVID-19 pandemic. Despite the different training arrangements, both versions shared a similar approach and training components. Training involved a mixture of lectures, self-work activities, observations, mindfulness sessions and role plays combined with self-reading and assignments. Whilst trainees received lectures from experienced OD practitioners about the philosophy, values and principles of OD, the perceived focus of the training was on self-work activities. Participants reported completing their own genograms, exploring how their own networks and family of origin had impacted them as a person and the way they relate to others and view the world. They also participated in mindfulness sessions and engaged in self-disclosure by sharing their reflections and personal experiences with others attending the training. More information about the training can be found on the provider’s website.^1^

### Experiences and impact of OD training

#### Transformative impact of training

Open Dialogue was perceived by participants as a radically different approach to mental healthcare compared to the usual approach they had been following in their clinical practice due to its emphasis on supporting networks through dialogue, non-directive practice and a flattened hierarchy. OD training seemed to reflect these values and was thus not perceived as a didactic process but more of an experiential introduction to a new culture, a new way of understanding mental health and as a prerequisite for the ‘cultural shift’ needed for OD practice:

“…it did prepare us with the basic tools and skills and whatever. But more so for me, the other thing it did was to try to make it a way of life. For me I see the Open Dialogue more as a culture, it’s a way of being that is just very different from how services are organised. So yeah from that point of view, you know it prepared me for a new culture, a new approach and a new understanding, a new way of relating to others and to the people who have used our services.” (P1)

Open Dialogue training was generally not perceived as a traditional form of training in which trainees learn a new approach and acquire new skills. Rather, training was sometimes experienced as a process of “*unlearning*.”

“I think training is the wrong word in a way, because it’s actually we’re not trained, it’s just, it’s stripping away if anything, stripping away how we were trained.” (P2)

Because of OD’s focus on ‘being with’ people in crisis, tolerating uncertainty, developing relationships and generating dialogue, participants discussed how they had to revisit their previous professional training as well as their own values and beliefs about mental healthcare. This was occasionally experienced as a challenge by some trainees who even initially thought of OD as “*some sort of cult*” (P3). However, as the training progressed participants acknowledged the value of the approach in being truly person-centred and empathic with the potential to transform mental healthcare services. One participant in a focus group mentioned how training helped them switch the focus in their practice *“from thinking to feeling*” whilst other participants in the group agreed with him.

“I was struck. It was a challenge to me, as somebody who had always paid a great, given great store to interpretation analysis as a route to knowledge and to performing well in the world, somehow what you knew and how well you analysed, how cleverly you could pull knowledge together into effective ways of action and that completely was thrown out the window. I learnt in the first day or two that what you interpret and analyse is actually, you have to go way, you have to get beyond that. It’s not about, it’s about not analysing, it’s about not knowing, it’s about not interpreting, it’s about not formulating and that was a huge kind of upturn in my whole understanding of what learning was about. That was a real challenge and a really interesting one.” (P4)

For this participant, the introduction to OD was an interesting challenge that contested their previous ideas about learning and led to personal transformation. This sense of a personal transformation as an outcome of OD training was a recurring theme amongst participants which was perhaps needed for participants to embrace the ethos of the approach.

When asked about their experiences of the OD training, the responses predominantly revolved around the impact of the training on a personal level and less on the acquisition of skills. Participants described their overall training experience as ‘transformative’, as a process that “*cannot not change you*” (P5), leading to radical changes in their worldview and the way they perceive themselves and relate to others. The transformative impact of the training was largely associated with its strong emphasis on self-work activities and reflective processes:

“I’ve changed, I’m massively changed as a person. And just acknowledging how important those people are and we did like the genograms and … just acknowledging how your family and your network and people around you have created who you are and I’ve always thought about that, but it’s never been really brought to the forefront of my mind.” (P2)

The participant highlighted how OD training changed them as a person, helped them realise the importance of their family of origin and how their networks have shaped who they are. Similarly, other participants discussed how increased self-understanding was one of the main outcomes of OD training. Although reflecting on their own networks and personal experiences was generally perceived as emotionally challenging, it enabled trainees to “*dig deep*” (P5), “*find their true voice*” (P6) and become *“more in tune*” (P7) with themselves. This process was considered empowering on a personal level and allowed some participants to make significant life changes (e.g., becoming vegan, re-evaluating relationships with significant others). At the same time, this enhanced self-understanding as a result of the training was perceived as a prerequisite for becoming an OD practitioner:

“The whole, you know Open Dialogue training is, a lot of it is being in tune with yourself, so you can support other people. You can’t support other people if you are not in tune with yourself, how can you be in tune with them?” (P7)

Self-awareness was thus viewed as a tool that practitioners could use in their dialogic practice, enabling them to better relate and empathise with service users. This outcome of the training arguably facilitated a shift in the focus of care from knowledge and expertise to emotional and relationship building abilities.

#### Team bonding

During the interviews, another frequently discussed outcome of the training was an increased sense of bonding between colleagues.

“It just was lovely to connect with people and you know you go into work and prior to Open Dialogue, people were just colleagues, you know you didn’t really share anything, they don’t know what you do at weekends, they don’t know who you sort of live with, unless you know you build a bit of a relationship with someone. But there was what 10 of us, 13 of us on the training together and I think we all connected really well, didn’t we and found out things about each other, which made us realise that they aren’t just a nurse, or a social worker, or a, they’re people, they are humans and we all probably share similar difficulties, similar highlights in life.” (P8)

Having to openly share reflections about their own personal experiences and influences of their networks highlighted the shared ‘humanness’ and vulnerabilities amongst trainees. Participants mentioned how they started seeing their colleagues as humans beyond their roles and professional titles and were thus able to better connect and relate to them. This was contrasted to the “*isolated way of working*” (P9) in TAU. Participants used the words ‘closeness’, ‘collectiveness’ and ‘togetherness’ to describe the team bonding that came as a result of having been through this intense learning experience together and having been able to support each other emotionally. Because most participants trained together with their colleagues from the same site, this increased sense of team bonding was viewed as essential for then moving on to form an OD team and work together in the co-facilitation of network meetings.

However, this sense of team bonding was not experienced by everyone. Participants who received the online version of the training mentioned that the human interaction and mutual support were missing in the online setting. Although participants engaged in self-disclosure and reflections with other trainees, they mentioned that they did not experience the sense of bonding reported by their colleagues that received the residential training. Similarly, two participants who were peer practitioners and attended the training without yet being a member of a team discussed experiencing feelings of loneliness and othering:

“I think because everybody had their own, you know they were in their own working groups, you know they’d come in teams from different NHS Trusts, um, yeah I did feel quite separate from everybody and again there was that sort of re-experiencing sort of a separateness and outsiderish feeling. I couldn’t understand why nobody wanted to, like I was sharing quite a lot you know in these essays and I thought, I don’t know I thought maybe that would create more connection. It was quite painful.” (P6)

#### Preparing for practice

Overall, most participants agreed that both residential and online training were successful in introducing them to the model and its principles whilst also equipping them with the essential skills needed to deliver OD. Role plays and observing network meetings were considered the most crucial aspects of the training in developing practical skills that would help them meet the fidelity criteria in practice.

“I just met wonderful people and learnt about a process that I really believed in, because it, I think the values behind it are ones that I have. So, it felt like everything coming together, yeah some coherence, you know I’m doing something good. I feel like I should say much more critical things, but for me it was really wonderful.” (P10)

However, some participants felt that there was room for improvement in terms of how well training prepared them for OD practice. One participant mentioned that more time and attention should have been given in role-plays, whilst someone else mentioned how training was so focused on self-work activities that the methods and theory of the approach were sometimes overlooked. This was echoed by another professional who believed that training was not adequate for professionals with no prior experience of working relationally with families.

“Personally, I didn’t feel that I had the right amount of experience, knowledge, bearing in mind I worked for the crisis team before doing this. So, I went in, did the intermediate level of family therapy, because it doesn’t quite prepare you for what you’re kind of holding, because you are not working really with one person, you’re working with their whole network, which is different, I think it’s quite different. Yeah so that’s my experience of it. It was good, but it wasn’t enough for me. You kind of get a taster of what it will be like and we get like the, okay you do this and you do that and you kind of have that first conversation and it gives you like the pointers if you like, to kind of start.” (P11)

This is not surprising considering that the year-long training participants received is considered a foundation training for Open Dialogue. However, most participants in this study had the chance to either offer network meetings in-between the residential weeks or soon after completing the training. This combination of training and hands-on experience was considered vital in gaining confidence in dialogic practice, especially for trainees that joined colleagues who had completed the training.

“Yeah, I think it does prepare you, you have to do the training to understand it all don’t you, you have to but the fact that I am actively in Open Dialogue sessions and doing the training, it’s like a lightbulb, oh okay I see it now.” (P12)

In terms of how well the online training prepared professionals for OD practice, the consensus seemed to be that the training had been adapted well but it was perhaps not as immersive as the residential version. A team manager (P3) believed that remote training required a higher level of “*intensity and focus*” by trainees in order to achieve the cultural shift needed for dialogic practice. In addition, two participants mentioned that practitioners who had completed the online training seemed less confident in delivering OD compared to those who had done the residential training. However, because participants who completed the training during the pandemic joined already established teams that were delivering network meetings, they had the chance to practice OD whilst training which helped with their immersion in the approach.

### Developing as an OD practitioner beyond training

The findings presented in the first theme indicate that formal training was overall successful in introducing trainees to the ethos and skills of OD, especially when trainees had the chance to practice their learning in network meetings. However, participants believed that OD is not just a technique that participants could simply incorporate into their practice after the training. Instead it was viewed as a way of being and relating to others, a different culture they had to invest time in:

“It takes time to embed yourself into a kind of a culture.” (P13)

“Open Dialogue is very much around a culture change and when it comes to the NHS you can’t shift a culture from doing bits of training.” (P14)

Participants believed that becoming an OD practitioner required ongoing learning beyond the formal training and a continuous commitment to the values of OD with even the most experienced practitioners mentioning that they are still learning years after the training.

“So it felt a kind of immersive experience (the training) was key and it does feel like we are learning, it’s now 17, 18, 19, 20 21, it’s the fifth year and I still feel like we are learning so much every InterVision, so it feels like very much, we are still very much at the beginning. It’s not in a way which feels uncomfortable, rather it’s okay to be learning.” (P15)

Viewing the process of becoming an OD practitioner as an ongoing learning curve beyond formal training, this theme investigates the facilitators and barriers in this process as discussed by the participants.

#### Facilitators—what helps in the process of becoming an OD practitioner

Ongoing engagement with OD through facilitating network meetings was considered the main avenue for learning and becoming more familiar with the approach. Participants gave accounts of how they learn through every network meeting they deliver and through every network they work with:

“But it’s like anything, I think you can be trained in anything and you can sit and learn how to do something, it’s putting it into action that actually makes you really get to grips with it, isn’t it? It’s the network meetings that have trained us, me personally.” (P2)

Apart from ongoing practice through facilitating network meetings, InterVision (a form of weekly dialogic and reflexive team supervision) was also perceived as a facilitator in the process of becoming an OD practitioner. Apart from it being a dedicated time for reflective practice and mutual support, InterVision provided a space for participants to revisit the values and ethos of OD and learn from each other’s practice and reflections.

“I think one of the key things when it works well, which is really important in bridging the gap between the training and the doing is the staff supervision that we have, you called it InterVision, because without that there is no other format we can discuss in dialogue.” (P16)

#### Challenges to becoming an OD practitioner: transitioning from training to practice

Despite the overall positive experiences of the formal training and the commitment of participants to dialogic practice, several systemic/organisational factors often challenged participants’ ability to fully embody the principles of OD in their service provision.

Most participants in this study were practising OD whilst also having a caseload where they offered TAU. The TAU approach has fundamental differences with OD due to its emphasis on a biomedical perspective that promotes diagnosis-led practice, medication-focused treatment, risk-assessment and was overall considered less person-centred. Having to switch between TAU and OD approaches in their practice was a challenge for some participants, especially when OD was only a fraction of their overall workload.

“Having to switch between the two different mentalities is very difficult, the two different cultures, the two different ways of working, very difficult. I found myself and I’m not sure whether it’s, I’m not sure why and I've been wondering why, I don’t know if it’s the further I get away from my training, I've noticed myself becoming less and less dialogical as time goes on. I don’t know whether that’s because I’m spending time in both teams and my Open Dialogue perspective is slowly becoming diluted. But it feels difficult to not become diluted.” (P14)

This indicates a potential gradual waning influence of training for some participants who had to continue delivering TAU alongside OD. Besides the fundamental differences between the two approaches, participants also discussed the challenge of introducing a novel approach such as OD in an organisation that is characterised by a ‘TAU culture’ with a greater emphasis on biomedical management of healthcare and intolerance to the uncertainty about risk.

“Staffing is shared across both teams and inevitably if there’s something going on in one team whether it’s resources or something else, it impacts on the OD team and more importantly the culture of the team, the two teams seep, you know there is a little bit of seepage across that interface in terms of the culture. More so from TAU over into OD, because that’s the, that’s what we’re surrounded with in the organisation, it’s a harder force to resist in terms of becoming less dialogical in our work.” (P17)

There was generally a sense from participants’ accounts that remaining faithful to the model was more challenging when service and trial demands were increased. For example, in some sites, having two OD practitioners facilitating each network meeting was not possible due to capacity issues. This difficulty in resisting the ‘TAU culture’ seemed to be even more prominent for smaller OD teams. Participants discussed how a particular crisis team only trained a couple of people to form a part of the overall OD team which was proven to be inadequate for the cultural shift needed for dialogical work. These trained practitioners had to deal with the increased pressure of crisis work and did not have managerial support for dialogical practice which led to a gradual decline in their interest of offering network meetings.

Other participants mentioned that in times of increased pressure on the service, such as during the COVID-19 pandemic, InterVision was impacted. This differed across sites, with some sites cancelling InterVisions, whilst in other sites these sessions were not used as a reflective space but were instead used to discuss caseloads, targets and fidelity criteria, an approach which did not reflect the ethos of OD and did not contribute to the development of the practitioners.

Besides the cultural influences of TAU and restrictions related to resources, participants reported that they also have to adhere to professional and organisational standards, policies and procedures which were often not aligned with the OD values.

“We still have to adhere to policies and procedures of the organisation that we are working for. We still have to work along the guidelines of our own professional bodies. So, there are certain standards, there are certain things that we have to adhere to as well to keep things safe, to keep you know. So, when it comes to risk, or safeguarding, or something like that we may have to make decisions that are not maybe discussed with the service user, or it doesn’t have full participation with them, if that, if you understand. So, there might be times when we do have to step out of a dialogical role and pursue a more treatment as usual’ sort of process.” (P9)

On such occasions, the principle of tolerating uncertainty and other elements of OD such as transparency and shared decision-making were undermined. However, despite having to sometimes deviate from OD, participants’ enthusiasm for the model helped them maintain their focus and motivation in being “*as dialogic as possible*” (P18). Altogether, the organisational factors discussed above could be considered as a barrier to participants’ learning curve of becoming an OD practitioner. This left some participants wondering whether bridging the theory-practice gap was an element of training that was missing, whilst others discussed the need for OD to be supported organisationally for it to be sustained.

“We don’t want to be surviving, we want to be sustaining an Open Dialogue model and it feels like sometimes our team feels at crisis point in terms of the resources that we have to operate in this way, which we would all want to operate in. So, it’s like for me bridging the theory of training, the theory, there’s a lot of practice within that, into the real world and the real demands, is a real challenge. There’s been a lot of changes, so how, and that’s something working in the NHS generally is about how is a good idea sustained, it needs to be nurtured from somewhere.” (P16)

## Discussion

This study aimed to examine the experiences of becoming an OD practitioner amongst practitioners who were part of the ODDESSI trial through individual, joint and focus group interviews. Our analysis yielded two main themes. The first theme focused on the transformational impact of formal training on trainees’ worldviews, relationships and preparedness for dialogic practice. The second theme explored the facilitators and barriers in transitioning to, and developing dialogic practice. In this section, our results are discussed through a Transformational Learning lens. Transformational learning has been applied across different contexts of adult learning including healthcare (Phillipi, 2010). The theory has been considered appropriate to inform and describe models of medical (Van Schalkwyk et al., 2019) and nursing education (Tsimane and Downing, 2020) and has also been used to illustrate the process of training in a psychotherapeutic approach (Watkins, 2022).

Disorienting dilemma has been defined as the first stage of transformational learning (Mezirow, 2000) which occurs when learners encounter new knowledge that does not fit or contradicts their pre-existing meaning, perspectives and previous learning experiences. Experiencing a disorienting dilemma is considered the catalyst for transformational learning, but it can also be an unsettling, disruptive and anxiety-provoking experience for learners (Roberts, 2013). This has been documented by studies with trainee therapists who, in the initial stages of their training, have expressed feelings of insecurity, self-doubt and disorientation (Watkins et al., 2018). Apart from some sporadic comments of initial scepticism about the training and the approach (e.g., “it felt like a cult”), participants in this study mostly welcomed OD training as an introduction to a new culture. They quickly realised how radically different OD was compared to the way they had been working and some mentioned the challenge of shifting focus from interpretation and analysis to ‘feeling’ and ‘being with’ service users and their networks. However, there was a sense of overall readiness for change and participants quickly embraced the ethos of OD without the uncomfortable feelings often described in the second phase of transformational learning (self-exploration with feelings of guilt and shame). This can be explained by the fact that participants had either been ‘cherry-picked’ by their organisation or had chosen to be part of the trial because they believed that a change in services was needed and OD was considered to be aligned with their personal values.

The transformational impact of the training on a personal level was a recurring theme in participants’ accounts. This was associated with the training’s intense focus on self-work, reflections and group sharing. Through reflecting on their life experiences, their family of origin, their relationships with others and their values, participants gained a deeper sense of self-awareness, becoming conscious of what, within the transformational theory, Mezirow (1991) has described as previously undisputed assumptions and frames of reference. This increased sense of self-awareness can be considered transformative in itself (Jaakkola et al., 2022). It allowed participants to become more “in-tune” with themselves and use their self-understanding as a resource and as a guide to be able to better support and relate to others. Studies which have explored the impact of performing such self-work activities as part of family therapy or counselling training, yielded similar findings, with participants reporting personal growth (e.g., increased self-acceptance, healthier relationships), increased empathy towards their clients and development of systemic thinking (Pistole, 1997; Lim, 2008). Participating in these self-work activities was often emotionally demanding for participants in our study. However, the residential and group training environment enabled them to support each other, fostering genuine bonds through the mutual sharing of personal experiences and vulnerabilities. Forming trusting relationships with other learners can be fundamental in the transformational learning process, helping learners develop ‘relational knowing’ (Mezirow, 1991), express shared concerns inherent to change and offer nonevaluative feedback and support to each other (Eisen, 2001; Choy, 2009). Participants’ experiences with these aspects of the training seem to reflect stages 2–4 of transformational where learners move from self-exploration, to critical assessment and recognition of shared experiences (Mezirow, 2000).

Beyond self-work, during the training participants engaged in interactive lectures, workshops, role-plays, observed and participated in network meetings. These more instructive and practical components of the OD training are aligned with the transformational learning stages of acquiring knowledge and skills and exploring and trying out new roles (Mezirow, 2000). Through these, participants believed they were able to develop the knowledge and skills required to become an OD practitioner and gain practical experience. However, participants reported that, beyond skills acquisition, the most crucial aspect of training was actually ‘unlearning’. Unlearning can be defined as “the process of reducing or eliminating pre-existing knowledge or habits that would otherwise represent formidable barriers to new learning” (Newstrom, 1983, p.36). Open Dialogue was perceived as a fundamentally different approach to mental health care when compared to the one most participants had been previously trained in and were following in their clinical practice. Unlearning was thus viewed as essential in facilitating the cultural shift needed for them to be able to develop as OD practitioners. As a process, unlearning has been considered challenging but it can be facilitated in learning environments that are secure and foster group learning, introspection and total (not gradual) immersion to the new learnings (Magrath, 1997 as cited in Macdonald, 2002). The residential setting of the training which almost all participants were exposed to in this study arguably facilitated such a learning environment which could explain why participants did not generally view unlearning as challenging, at least during their formal training.

The final stages of transformational learning are ‘building competence and confidence’ and ‘reintegration’ (Mezirow, 2000). For participants in this study these stages can be reflected by their transition from formal training into practice. The process of becoming an OD practitioner was perceived as an ongoing learning curve extending beyond formal training and into real-world practice. Participants continued learning and gained confidence as OD practitioners via facilitating network meetings and incorporating their transformed worldviews and new skills in their clinical work. Their commitment to the ethos of OD and their regularly scheduled reflective meetings (InterVisions) acted as facilitators in these final stages of their learning.

Yet, despite participants’ efforts and enthusiasm, they were also faced with impeding factors. It has been argued that, especially for experienced professionals, unlearning can be a lengthy process which should be supported by organisations that aim at achieving transformational change (Macdonald, 2002). In this study, most participants’ unlearning process was challenged by still having to practice TAU and working in organisations characterised by an approach to mental healthcare which is fundamentally different to the ethos of OD. Although OD training was effective in initiating the process of culture change (at least within the OD teams that were formed), this process may not have been adequately supported on an organisational level. Lennon et al. (2023) argue that the implementation of humanistic approaches, such as OD, in conventional mental health services requires complex systemic change and adaptive leadership that is committed to change and managing the consequent organisational tensions. However, in our study, most of these small OD teams were formed to fulfil the needs of a trial and did not reflect an attempt to introduce OD as the dominant approach and culture across these organisations. An Australian study about the implementation of OD in a private healthcare setting yielded similar findings, with practitioners feeling discomfort in still having to follow a conventional model of care on top of their work in OD (Dawson et al., 2021). Participants felt that contradicting professional expectations, resource demands and hierarchical structures in the organisation could not allow OD to flourish, despite their personal enthusiasm and support for the approach.

### Implications

Our findings indicate that despite participants completing the year-long OD foundation training programme, they reported similar transformative training experiences to those reported by professionals who completed extended (two and three-year) OD training courses (Wates et al., 2022; Buus et al., 2022b). Training was successful in initiating a process of cultural shift and the impact on a personal level was considered essential for subsequent dialogic practice. However, echoing the feedback of participants, OD training programmes should potentially place more emphasis on the acquisition of skills and supporting trainees in their transition to practice. The findings presented in this paper suggest that transformational learning theory can be a useful model to inform the structure of OD training and to help educators better support trainees’ transition through the different stages of learning. Moreover, future research should examine whether there are significant differences in competence and preparedness for practice between practitioners who have completed foundation and extended training programmes in order to appropriately guide future implementation efforts. Nevertheless, based on the experiences of the participants, formal training was only considered to be the start of a lengthy process of becoming an OD practitioner and sustaining dialogic practice. Aligned with the conclusion of Buus et al. (2021) who conducted a scoping review of implementation efforts of OD globally, we believe that organisations who aspire to introduce OD to their services should be cognizant of the need to support practitioners by being committed and ready for change. This could include appropriately managing resources in a way that reflects the demands of dialogic practice (e.g., having two practitioners in every network meeting) and reviewing current policies and protocols which might contradict dialogic practice (e.g., emphasising the need for diagnosis and standardized risk-assessments). However, we understand that such radical changes in healthcare organisations could only be considered if OD shows promising clinical effectiveness via robust clinical studies.

### Limitations

One of the limitations of this study is that data came from a subset of interviews whose main focus was the overall experiences of practitioners in delivering OD. Although data presented here related to questions asked specifically around training experiences and transitioning to practice, our findings would be arguably more nuanced if the interviews had solely focused on these topics. For example, participants discussed the acquisition of skills vaguely without specifically mentioning essential skills for dialogic practice. Secondly, data were collected between June 2020 and April 2021. The experiences of trainees who have completed newer versions of the same training might be different and improvements in the programme might have been made. Nevertheless, the findings presented here reflect the experiences of OD practitioners who were part of the ODDESSI trial which will be useful in interpretation and contextualising the trial outcomes in the future. Finally, only a small minority of participants in this study received the online version of the training. Considering that this online version is still being delivered, we believe that more evaluative information from practitioners who attended this version of OD training would be merited.

## Conclusion

Our study showed that the one-year foundation training in OD was well-received by practitioners who perceived it as a transformative experience. Apart from equipping them with the essential skills and knowledge for dialogic practice, participants particularly commended training’s impact on a personal level. Being introduced to the ethos of OD, team bonding, increased self-awareness and ability in working relationally were some of the training outcomes that practitioners believed were essential in delivering the approach. However, becoming an OD practitioner was viewed as a longitudinal process beyond formal training that required ongoing engagement with the approach via practice and organisational support and flexibility. Despite participants’ enthusiasm and faith in the approach, their efforts in providing optimal OD care were sometimes undermined by organisational restrictions, limited resources and often having to deliver treatment as usual alongside OD.

## Data availability statement

The raw data supporting the conclusions of this article will be made available by the authors, without undue reservation.

## Ethics statement

The process evaluation received ethical approval from the Health Research Authority and the Health and Care Research Wales committee (Project ID: 259468). The studies were conducted in accordance with the local legislation and institutional requirements. The participants provided their written informed consent to participate in this study.

## Author contributions

EA: Conceptualization, Data curation, Formal analysis, Investigation, Methodology, Project administration, Validation, Writing – original draft, Writing – review & editing. TW: Conceptualization, Formal analysis, Funding acquisition, Methodology, Project administration, Supervision, Validation, Writing – original draft, Writing – review & editing, Data curation, Investigation, Resources. CM: Conceptualization, Data curation, Investigation, Methodology, Project administration, Validation, Writing – review & editing. KC: Project administration, Resources, Supervision, Validation, Writing – review & editing. SP: Conceptualization, Funding acquisition, Project administration, Resources, Supervision, Validation, Writing – review & editing.

## Footnotes

^1^ https://opendialogueapproach.co.uk/
