# Supplementary material for: Becoming an Open Dialogue practitioner: a qualitative study of practitioners’ training experiences and transitioning to practice
Source: Front Psychol. 2024 Aug 12;15:1432327. doi: 10.3389/fpsyg.2024.1432327 (PMC11345226; doi:10.3389/fpsyg.2024.1432327)
Supplement: Supplementary file 1 [file Table_1.DOCX]

*Data presented in this paper have been extracted from interviews with a broader focus on OD practitioners’ overall experiences of delivering Open Dialogue and facilitating network meetings (to be reported elsewhere). Below, you can see some of the questions that specifically relate to the scope of this study, practitioners’ experiences of OD training and transitioning to practice. Please note that because interviews were semi-structured, prompts were used to help participants further elaborate on their individual experiences.*

*All practitioners will have received OD training prior to starting work on the trial, can you tell me a little bit about the training programme you completed?*

*How well do you think the training has prepared you for delivering OD?*

*How did you experience transitioning from training to delivering OD within your service?*

*Since you also work across TAU teams, do you have a TAU caseload in addition to OD? How have you experienced this?*

*Can you talk to me about your experience of implementing OD within an NHS setting?*
